# Human papillomavirus vaccine coverage surveys in low- and middle-income countries: current efforts and future considerations for very young adolescents

**DOI:** 10.1136/bmjgh-2024-018731

**Published:** 2025-06-13

**Authors:** Lia Pak, Julia Rollison, Maya Rabinowitz, Laura J. Faherty

**Affiliations:** 1 RAND, Boston, Massachusetts, USA; 2 RAND Corporation, Arlington, Virginia, USA; 3 Department of Psychology, Yale University, New Haven, Connecticut, USA; 4 Maine Medical Center, Portland, Maine, USA; 5 Tufts University School of Medicine, Boston, Massachusetts, USA

**Keywords:** Vaccines, Global Health, Child health, Immunisation, Review

## Abstract

With the recent accelerated rollout of the human papillomavirus (HPV) vaccine in low- and middle-income countries (LMICs), there is a growing need for high-quality vaccination coverage measurement. Vaccine coverage surveys are a key avenue for collecting coverage data, but little is known about the current state of HPV vaccination coverage surveys of very young adolescents (VYAs)—those 10–14 years of age—in LMICs and methodological considerations for these efforts. Through an analysis of peer-reviewed and grey literature and a series of expert discussions, we identify promising approaches for these coverage surveys, such as when to sample from schools versus households and how to reduce recall bias. We also draw attention to the significant methodological gaps, such as a lack of research comparing the validity of vaccination status self-report by the VYA to a caregiver’s report. Next, we describe the status of coverage surveys, finding that most LMICs with the HPV vaccine included in their national programme have not conducted a nationally representative HPV vaccination coverage survey. We also describe four existing multi-country survey efforts that include HPV vaccination coverage questions. Finally, we discuss promising approaches to strengthen survey measurement of HPV vaccination coverage among VYAs. Our findings lay the groundwork for stakeholders to expand HPV vaccination coverage measurement for VYAs in LMICs, a necessary component for reducing global HPV and cervical cancer burdens.

**SUMMARY BOX**

- Despite the accelerating rollout of the human papillomavirus (HPV) vaccine in low- and middle-income countries, vaccination coverage measurement is limited.
- Evidence is sparse on key methodological considerations for surveying very young adolescents (VYAs), the age group that primarily receives the vaccine.
- We identified four multi-country survey efforts with questions about HPV vaccination status, but there are currently no HPV vaccination coverage questions primarily administered to VYAs.
- Depending on measurement goals and available resources, HPV vaccination coverage measurement among VYAs could be strengthened by expanding existing multi-country surveys to include adolescents younger than 15 or by developing new national surveys aimed at VYAs.

## Introduction

 The human papillomavirus (HPV) vaccine is effective in preventing HPV infection, the primary cause of cervical cancer. With around 350,000 annual deaths due to cervical cancer, mostly in low- and middle-income countries (LMICs),^1^ high levels of HPV vaccination coverage can greatly improve global health.^2 3^

The WHO recommends the HPV vaccine for girls 9–14 years old, which typically involves two or three doses but is also effective as a single dose.^4^ Among countries that have added the HPV vaccine to their national immunisation schedules, vaccine delivery strategies vary.^5 6^ School-based delivery is often used to reach adolescents, but some countries use health facilities as primary or supplementary delivery locations. Some countries have begun providing the HPV vaccine to boys, but settings with limited vaccine supply or infrastructure prioritise vaccinating girls. National programmes may target different ages within the 9- to 14-year age group, and some offer secondary prevention or catch-up vaccines for adults.

While the HPV vaccine has been available since 2006, its introduction in LMICs has lagged behind high-income countries (HICs). Within the first 10 years of the vaccine’s introduction, 80% of HICs introduced the HPV vaccine, but less than 20% of LMICs had done so.^5^ However, momentum has been increasing. In 2019, 17 new LMICs introduced the vaccine. Then, in 2020, WHO established a 2030 goal of fully vaccinating 90% of girls before age 15; at the time, only 13% of girls had received all vaccine doses before age 15.^7 8^ However, this target is becoming increasingly possible, with 33 additional countries introducing the vaccine between 2020 and 2023.^7^ Additionally, WHO recommendations for single-dose schedules have the potential to improve access and increase rates of full vaccination.^9^

With this accelerating global rollout, there is also a growing need to collect data on vaccination coverage to assess programme implementation, identify regions or countries that require additional vaccination programming, and more broadly, monitor adolescent health at the national and global levels. In fact, the Global Action for Measurement of Adolescent (GAMA) health initiative included HPV vaccination coverage as one of 47 important indicators of adolescent health.^8^ However, varied delivery settings and populations make it difficult to standardise measurement, adolescents pose different ethical and logistical data collection challenges than other age groups, and stigma around the HPV vaccine’s perceived connection with sexual activity can influence people’s willingness to share vaccination status.^6 10^ Most HPV vaccine coverage estimates in HICs rely on administrative data such as national immunisation registries, but administrative vaccine data in LMICs is often less reliable.^11^ WHO and UNICEF national coverage estimates rely on a combination of administrative and survey data, but these face quality and standardisation challenges and could be improved with more high-quality coverage surveys.^5^ Additional standardisation and documentation of current survey efforts are crucial for evaluating the success of the HPV vaccine roll-out, monitoring coverage rates and assessing targeted interventions.

In 2024, the Gates Foundation (hereafter, ‘the foundation’) funded our research team to conduct a landscape review of HPV vaccination coverage surveys of very young adolescents (VYAs)—those 10 to 14 years of age—in LMICs. Recognising the gap between the rapidly evolving HPV vaccine roll-out and lagging coverage data, the foundation was particularly interested in promising avenues to advance survey measurement efforts for this age group, which is often excluded from large-scale surveys but is the primary recipient of HPV vaccines.

To inform discussions on future directions for expanding HPV coverage measurement among VYAs, we summarise (1) methodological insights from the literature and experts in the field on promising practices and considerations to obtain more valid and reliable data on VYA HPV vaccination status, (2) the global landscape of existing HPV vaccination coverage surveys and (3) promising approaches to expand survey measurement of HPV vaccination coverage.

## Information sources and our synthesis process

To inform methodological insights, we began with a review of peer-reviewed and grey literature, as well as existing documentation shared by the foundation and their partners. The peer-reviewed search was initially targeted at priority countries identified by the foundation. The priority country search identified 74 articles. 36 articles were abstracted following deduplication and excluding studies published before 2014, those not related to HPV vaccination surveys, and those not involving VYAs. Included studies were reviewed by two study team members, with the first five articles abstracted by both. There was high initial alignment, so the remaining articles were abstracted by only one team member.

This search was followed by targeted searches on three questions of interest to the foundation about VYA survey measurement methodology: (1) is it preferable to use school- or household-based sampling to generate representative survey samples? (2) is it better to survey the VYA or their caregiver about HPV vaccination status? and (3) what strategies can reduce recall bias in this context?. A total of 16 articles were identified through the targeted methods search and, after excluding those published before 2014, 10 were reviewed. Relevant text was extracted by Elicit, an AI research assistant tool,^12^ with review and additional details added by the same two team members.

We supplemented the literature scan with semi-structured discussions with seven experts on VYA measurement and HPV coverage surveys in LMICs. These experts were identified primarily based on recommendations from the foundation and their partners. We took near-verbatim notes during the discussions, and recorded with permission to supplement notes as needed.

We conducted a parallel effort to document publicly available information on the status of HPV vaccination coverage surveys by country. We leveraged the WHO’s HPV dashboard for 196 countries^7^ which details the status of vaccination coverage programmes and delivery strategies and used information from our discussions and existing documentation to fill in details. For countries where we had no existing information, we conducted Google and Google Scholar searches—country by country—using search terms including, “HPV vaccine coverage”, “HPV vaccine survey” and “HPV vaccine coverage survey”. While our focus was on nationally representative surveys, we also noted details of smaller, one-off coverage surveys identified through these searches.

We organised and synthesised findings across data sources using an Excel workbook that captured: (1) large multinational survey platforms including HPV vaccination coverage questions; (2) survey studies in priority countries; (3) methods literature with details on key findings; and (4) country-level HPV survey status. Finally, notes from expert discussions were entered into a separate Excel file which allowed for comparison and synthesis by topic (columns) and documentation of each source (rows). All data collection and rapid analysis occurred in spring and early summer of 2024.

## Methodological considerations

Through the rapid analysis of literature and expert discussions, we identified insights on methodological considerations and promising approaches to obtain valid survey data on VYA vaccination coverage and areas requiring future research. We focus on three areas of interest: ideal sampling strategies, surveying VYAs versus their caregivers and minimising recall bias. Characteristics of the articles reviewed are shown in Table 1.

**Table 1:** Characteristics of reviewed articles about human papillomavirus (HPV) vaccination coverage surveys and very young adolescent survey measurement

	Priority country articles (n=36)	Methods articles (n=10)
WHO region*		
Africa	25	1
Americas	1	6
Eastern Mediterranean	0	0
Europe	0	0
South-East Asia	10	0
Western Pacific	0	1
Multiple	0	2
Article type		
Survey analysis	36	7
Literature review	0	3
Sampling setting		
Household	11	1
School-based	23	1
Healthcare facility	1	5
Multiple settings	1	0
N/A (not survey)	0	3
Data collection method		
Self-administered survey	22	4
Other-administered survey/interview	12	3
Multiple methods	2	0
N/A (not survey)	0	3
Respondent type		
Adolescent	13	2
Caregiver	21	4
Both	1	1
N/A (not survey)	0	3

* Priority country articles were targeted to specific countries of interest, as identified by the foundation, and were not intended to be representative of HPV vaccination survey efforts.

### Sampling strategies

We did not identify any peer-reviewed studies focused on sampling approaches for HPV vaccination coverage surveys in LMICs. Experts we interviewed suggested that the sampling strategy and target population should depend on HPV vaccine delivery.^13^ If the vaccine is delivered through schools, or if school enrolment is high among the target population, then school-based sampling may be appropriate. As noted in WHO’s reference manual for conducting vaccine coverage cluster surveys, the need to sample out-of-school girls depends on whether the survey’s goal is to estimate the success of a school-based delivery strategy or if it is to estimate population-wide coverage.^14^ Among the multi-country HPV surveys we examined, described more below, sampling strategies varied. USAID’s Demographic and Health Survey (DHS) programme and UNICEF’s Multiple Indicator Cluster Surveys (MICS) sample at the household level, while the WHO’s Global School-Based Student Health Survey (GSHS)^15^ typically uses multi-staged sampling at the school and classroom levels.^15^,^18^

### Surveying VYAs versus caregivers

We also did not identify any studies in LMICs that directly compared VYA HPV vaccination reports to their caregiver’s. The closest approximation was a limited DHS pilot with older adolescents conducted as part of national survey in Uganda. The pilot found that adolescents between the ages of 15 and 17 reported vaccination status with greater certainty than mothers of girls aged 9–14, but full results are not publicly available.^19^ There is also little evidence on survey response validity from HICs, as most research is focused on comparing parent report to administrative data.^20^,^22^ One exception is a US-based study in several urban adolescent clinics that found that mothers were slightly more accurate at recalling their daughters’ vaccination status than their 15- to 17-year-old daughters, but it is unclear if these findings would hold among younger adolescents or in countries with school-based vaccination.^23^ In short, there is no definitive evidence to support asking either the VYA or their caregiver about HPV vaccination coverage. However, in cases where HPV vaccination occurs in school (as is often the case in LMICs), experts agreed it is preferable to ask the adolescent directly, as caregivers may be unaware that their adolescent was vaccinated.

### Recall bias

The literature on strategies to reduce recall bias in this context was very limited. Some studies in HICs discussed vaccine recall variation by respondent characteristic (eg, higher recall among mothers than fathers), but few shared specific strategies to improve recall, and in general, may not generalise to LMICs due to differences in vaccine delivery strategies.^21 22^ Thus, recommendations on reducing recall bias came primarily from experts who stressed the importance of asking about vaccination coverage as soon as possible after vaccination. They noted that providing background information (eg, where on the body the vaccine would have been given, the brand name) may assist with recall, but cautioned against providing too much information to avoid introducing response bias for any knowledge or attitude questions on the survey.

## Landscape of HPV coverage surveys

### Prevalence of national coverage surveys

As of summer 2024, there were 41 LMICs that included the HPV vaccine in their immunisation schedule, either at the national or sub-national level.^7^ Of these 41, we found evidence that 16 countries had planned or completed at least one coverage survey, 6 of which were at the national level. All 6 used HPV modules within existing multi-country survey platforms. Another 6 of the 14 had national coverage surveys planned for 2024 or 2025. The remaining 4 countries conducted sub-national surveys, typically as one-time research, rather than government-led measurement efforts. Of the 25 countries that had not conducted coverage surveys, 5 only added the HPV vaccine to their national schedule in 2023.

For completeness, we also examined HPV vaccination coverage surveys in HICs and found none that surveyed VYAs directly to produce coverage estimates, instead relying on caregiver report or a combination of caregiver report and administrative data.

### Multi-country survey efforts

In this landscape review, we identified four multi-country survey efforts in LMICs that include questions about HPV vaccination status. The two largest surveys, DHS and MICS, collect nationally representative data on a wide range of population metrics using household surveys. They are implemented similarly, with base questionnaires that are administered consistently in participating countries and a series of optional modules. Both DHS and MICS have recently added modules on HPV vaccination. DHS piloted this module in 2021 to add to DHS-8 and MICS added it to MICS-7 in 2023.^19 24^ These modules are very similar, with both asking 15- to 17-year-old girls if they have received the HPV vaccine, the number of doses received, type of facility where they were administered the vaccine and if they were given a vaccine card.

WHO’s Expanded Programme on Immunisation (EPI) cluster survey is another population-based household survey that produces national immunisation coverage estimates. Unlike MICS and DHS, EPI is not a survey platform with prepared questionnaires and infrastructure, instead, it serves as a specific methodological approach with detailed implementation guidance.^14^ While EPI has been used to assess HPV vaccine demonstration projects as early as 2008,^25^ few countries have implemented it after adding the vaccine to their national schedule. Finally, the GSHS, also led by WHO, added five optional HPV vaccination questions in 2021.^26^ However, it is unclear how many and which countries have used these optional questions to date.

Each survey’s HPV vaccination measures differ in their administration, but only GSHS directly surveys 10- to 14-year-olds who are eligible for the vaccine. Table 2 contains additional details on each survey.

**Table 2:** Multi-country surveys with questions on human papillomavirus (HPV) vaccination

Survey	Details on HPV vaccine coverage measurement
**Demographic and Health Survey (DHS**)Led by ICF (and primarily funded by USAID), these surveys focus on nationally representative data on topics such as family planning, gender, child health, etc. There have been over 400 surveys administered in ~90 countries	DHS-8 collects data from adolescents, ages 15–17, as part of a separate optional module, to ask about vaccination Questions focus on receipt of vaccine; whether there is a vaccination card; number of doses; and location of vaccine receipt A few DHS surveys previously contained a question about having vaccination cards for HPV and used that as a proxy for coverage DHS uses enumeration areas to sample
**Multiple Indicator Cluster Surveys (MICS**)Led by UNICEF, MICS is a cross-country survey that generates data on various well-being indicators for children and women. It has been fielded for almost 30 years and implemented in ~120 countries	MICS-7 collects data from adolescents, ages 15–17, as part of a separate optional module to assess HPV vaccination coverage Questions focus on receipt of vaccine; whether there is a vaccination care; number of doses; and location of vaccine receipt (adapted slightly from DHS) One child is randomly selected from each household
**Expanded Programme on Immunisation** (**EPI) cluster surveys**Led by WHO, these surveys have focused on capturing data on immunisation coverage across a variety of countries; WHO maintains a reference manual for these surveys	These are population-based household surveys adapted from WHO guidelines on immunisation surveys Sampling approaches varied by country based on target population characteristics and vaccination coverage strategy Respondents were any adults that could verify a girl’s HPV vaccination status
**Global School-Based Student Health Survey (GSHS**)Led by WHO, modelled after CDC’s Youth Risk Behaviour Surveillance System, these self-administered surveys are administered in schools	The GSHS is fielded to adolescents 13–17 years of age in schools The core survey has been implemented in well over 100 countries since launching in/around 2000 Survey consists of core modules (countries must choose 6 of 10); optional modules can be added but inclusion is highly variable and limited In 2021, a ‘core expanded’ optional module on HPV was added, consisting of five optional questions (including one on the number of doses received); questions can be asked individually or as a set

## Looking ahead: expanding HPV vaccination coverage surveys

In this analysis, we characterised the current state of HPV vaccination coverage surveys and described key methodological considerations for surveying VYAs about their HPV vaccination status. Overall, there are few LMIC country-specific or multinational coverage surveys, and none that solely focus on VYAs, making it difficult to compare methodologies. Additionally, little can be drawn from HICs, both because of their inherent differences in vaccine administration and infrastructure and because few HICs conduct national HPV vaccination coverage surveys. Still, some promising practices emerged from the literature and conversations with experts. First, coverage surveys should focus on the populations targeted for the vaccine in national immunisation schedules. Second, if the vaccine is administered in schools, it is preferable to survey adolescents about vaccination status, rather than their caregivers and sufficient to conduct school-based sampling. However, if not administered in schools, it is important to consider other techniques, such as household-level sampling or street intercept models, to capture out-of-school youth. Third, questions on immunisation should be asked as close to the time of immunisation as possible. Finally, more methodological research is needed to compare VYA to caregiver recall and identify methods of improving the validity of vaccination. This may involve further validation of self-report accuracy across different populations or conducting technology-based survey administration.

One goal of this landscape review was to identify promising approaches to expand and maintain ongoing measurement of HPV vaccination coverage through surveys. One possible approach is to expand existing platforms, such as DHS, MICS or GSHS, while another approach is to support country-led and country-specific surveys. Both options have benefits and drawbacks. DHS and MICS have existing infrastructure, with regularly conducted base surveys implemented in many countries and existing HPV questionnaires. However, neither DHS’ nor MICS’ HPV modules survey VYAs, creating a lag in coverage data. GSHS surveys adolescents as young as 13, allowing for data closer to the age of vaccination. However, GSHS is implemented in fewer countries than DHS or MICS and has no publicly available information on testing and validation of its HPV-related questions. Additionally, GSHS is only administered in schools, so while it may be appropriate for countries with school-based vaccination, it is less feasible for collecting representative data in other countries.

It is also important to consider that the existing platforms’ optional HPV modules still require tailoring to country-specific factors such as vaccine delivery setting, vaccine formulation and target population(s). Country-specific surveys have the benefit of being able to be tailored to local contexts from inception. However, too much variation in questionnaires and sampling can limit cross-country comparisons and their feasibility depends on existing survey infrastructure, and commitment and funding for ongoing measurement. Measurement guidelines such as the WHO EPI survey manual and GAMA indicator of adolescent health could aid with standardisation for countries that develop their own surveys. However, we found no evidence of existing independent, country-specific, national coverage surveys in LMICs, which could indicate that such efforts are not currently feasible.

Ultimately, the viability of options to expand HPV vaccine coverage measurement depends on measurement goals. For example, if the intent is to capture information on national or subnational coverage to inform more near-term programme improvements (eg, increase vaccination promotion efforts to VYAs who did not receive an HPV vaccine between ages 9 and 11), no existing platforms directly survey VYAs and would meet this need. Thus, it may be necessary to develop new surveys, based on the learnings from existing surveys and tailored to specific information needs. If the intent is to perform a longitudinal assessment of HPV vaccination coverage among VYAs, then DHS and MICS may be viable options to explore. However, the future of these platforms is uncertain given rapidly changing global health funding.

This landscape review had certain limitations. First, our review of the literature was not systematic. We did not aim to review all literature on HPV vaccination coverage surveys and primarily used targeted searches to identify current surveys and answer key methodological questions. Thus, we may have missed relevant information. However, our focus on specific methodological questions was informed by the foundation and its partners, ensuring our searches and synthesis addressed areas relevant to stakeholders. Second, we did not speak with health officials from LMICs. Our expert discussions included individuals from five countries, many of whom had on-the-ground survey administration experience in LMICs, but it is possible that individual countries have planned or conducted national surveys that are not publicly available. Finally, the DHS, MICS and GSHS HPV questions we reviewed were introduced recently and have only been implemented in a few countries. Thus, we had limited information on their successes and challenges.

## Conclusions

With the accelerating roll-out of the HPV vaccine in LMICs, there is a clear need for coordinated and robust vaccine coverage measurement. Informed by research literature and experts’ on-the-ground experiences, this analysis provides important insights into the current state of HPV coverage surveys and methodological considerations for future efforts. While large survey platforms like DHS and MICS are well-documented, this review is the first to map existing multi-country survey efforts in LMICs, compare their pros and cons for strengthening HPV vaccination coverage measurement, and discuss them in the context of VYA survey methodology. Continued research into best practices for collecting reliable data on VYA HPV vaccination and concerted approaches for expanding survey efforts in LMICs will be crucial for improving measurement and ultimately increasing global HPV vaccination coverage.

## Data Availability

Data are available upon request.
